# Diagnosing rare intraductal biliary neoplasms – Intraductal papillary neoplasm of the bile duct: A case report with typical imaging findings

**DOI:** 10.4102/sajr.v26i1.2387

**Published:** 2022-04-29

**Authors:** Saumya Pandey, Nitin Agarwal, Vidushi Gupta, Ashok Sharma, Anil Aggarwal, Sunita Gupta, Ram Krishan

**Affiliations:** 1 Department of Radiodiagnosis, Govind Ballabh Pant Institute of Post Graduate Medical Education and Research, Delhi University, New Delhi, India; 2 Department of Gastrointestinal Surgery, Govind Ballabh Pant Institute of Post Graduate Medical Education and Research, Delhi University, New Delhi, India; 3 Department of Pathology, Govind Ballabh Pant Institute of Post Graduate Medical Education and Research, Delhi University, New Delhi, India

**Keywords:** intraductal neoplasms, solid-cystic, papillary growth, biliary dilatation, mucin production, communicating, hyperenhancing

## Abstract

Intraductal papillary neoplasm of the bile duct (IPN-B) is a rare preinvasive intraductal pathology of the biliary tract. It should be differentiated from other more common benign or malignant causes of biliary obstruction and dilatation such as calculi or cholangiocarcinoma because the management and prognosis of this condition differs significantly. This case report describes a case of IPN-B in a 45-year-old female patient who presented with non-specific complaints of chronic abdominal pain without jaundice for three months.

## Introduction

Intraductal papillary neoplasm of the bile duct (IPN-B) is a rare intraductal pathology of the biliary tract, accounting for 10% – 15% of biliary tract neoplasms.^1,2^ Classically, the term has been used to describe lesions that present as an intraluminal papillary growth around a fibrovascular core, with evidence of intracellular and/or extracellular mucin. Excessive mucin production often causes dilatation of the biliary tracts.^3,4^ While 5% – 20% patients may be asymptomatic, most present with non-specific complaints of chronic abdominal pain, intermittent jaundice and fever.^2,3^ According to the WHO classification of intraductal pathologies found in the biliary system, IPN-B is a type of preinvasive intraductal neoplastic entity, which is often a precursor lesion to invasive adenocarcinoma.^2,5^ It is important to rule out commoner causes of biliary duct dilatation, including stones and more sinister neoplastic aetiologies like invasive cholangiocarcinoma (intraductal type) as they carry different management and prognosis. We herein report a rare case of incidentally detected, intrahepatic IPN-B causing cystic dilatation of the left ductal system.

## Case report

A 45-year-old female patient presented to our gastroenterology outpatient department with complaints of a ‘dragging’ type, chronic abdominal pain for the past three months. There was no accompanying history of weight loss, yellowish discoloration (jaundice) or fever. There was no history of pruritis, loss of appetite or discoloration of stools. On clinical examination, the presence of hepatomegaly was observed; however, there was no evidence of a gall bladder lump on palpation.

Laboratory investigations revealed mild elevation of the serum total (~1.5 mg/dL) and direct bilirubin (~0.9 mg/dL) levels. Hepatic aminotransferase enzymes also showed some elevation (alanine aminotransferase [ALT]~100 IU/L; aspartate aminotransferase [AST]~90 IU/L). There was also a mild elevation of the alkaline phosphatase (ALP) levels (~198 IU/L). However, tumour markers, carcinoembryonic antigen (CEA) (~1.5 ng/mL), alpha-foetoprotein (AFP) (~13 ng/mL) and carbohydrate antigen (CA) 19–9 (~18 IU/mL) were well within normal limits. There was no evidence of leucocytosis (Total leukocyte count [TLC]~ 6000/µL) or eosinophilia (Differential leukocyte count [DLC] revealed eosinophils < 5%).

Suspecting a low-grade biliary obstructive condition, the patient was initially referred for abdominal ultrasonography (USG). Trans-abdominal USG of abdomen revealed a large, well-defined cyst in relation to the left segmental bile ducts, which appeared to be dilated. An irregular, isoechoic, papillary mural thickening was observed arising from one of the cyst walls, projecting into the lumen. No significant vascularity was appreciated on colour Doppler scans. However, due to the large cyst size and limited acoustic window, biliary connection with the cyst could not be ascertained. Given the solid-cystic nature of the lesion at USG, hydatid (immunoglobulin G [IgG] titre-0.3; normal < 0.9) and amoebic serology (IgG titre-0.47; normal < 0.9) was requested, which were both non-reactive.

**FIGURE 1:**
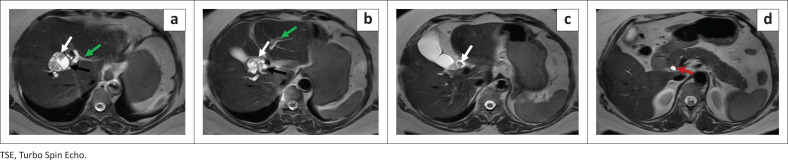
T2W–TSE axial sections showing cystic dilatation (black arrow) at the primary confluence with internal, mural-based, frond-like hypointense contents (white arrow), extending to the common hepatic duct/CBD. Upstream dilated biliary radicles (green arrow) are observed with a mildly dilated CBD distally (red arrow).

**FIGURE 2:**
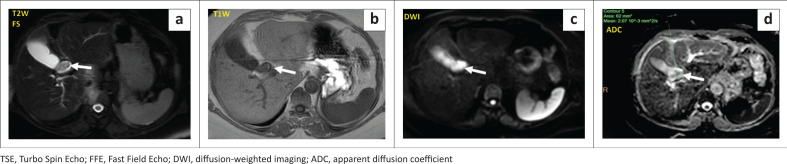
Axial T2W–TSE (a) and T1-FFE (b) sections demonstrating an iso to hypointense mural-based growth (white arrow) projecting into the lumen of the common hepatic duct or CBD. High signal is observed within the lesion on the axial b-1000 DWI image (c) with low signal on the corresponding ADC map (d).

**FIGURE 3:**
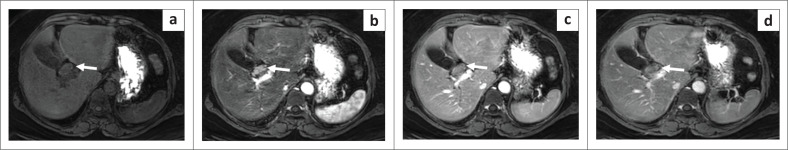
Dynamic post-contrast axial sequences revealing iso-intensity of the tumour (white arrow) on noncontrast (a), hyperenhancement on the late arterial phase (b), reduced hyperintensity on the porto-venous (c) and delayed (d) phases.

**FIGURE 4:**
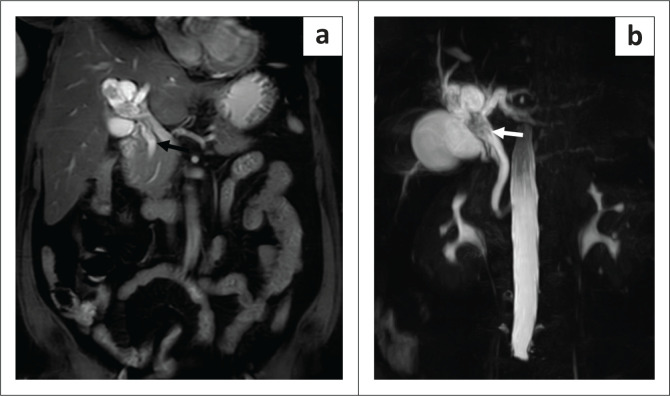
(a) Balanced Turbo Field Echo (BTFE) coronal section showing the dilated downstream common hepatic duct and CBD (black arrow), separate from the lesion at the primary confluence. The site and extent of the intraductal tumour is best seen on the volumetric MIP of the magnetic resonance pancreatico-cholangiography (b), indicating extension of the tumour into the common hepatic duct (white arrow) with upstream and downstream ductal dilatation.

Due to the limited information on USG and to further evaluate the status of the biliary radicles, contrast-enhanced magnetic resonance imaging (CE-MRI) of the upper abdomen was performed. T1-weighted imaging demonstrated a well-defined cyst at the hilum with an isointense frond-like soft tissue lesion projecting into the cyst and extending into the common hepatic duct (CHD)/common bile duct (CBD). Upstream bilobar intrahepatic biliary radicles appeared to be mildly dilated. T2-weighted and magnetic resonance pancreatico-cholangiography (MRCP) sequences revealed the lesion to be a cystic, focal expansion of right hepatic duct and primary confluence with upstream minimal biliary dilatation. Downstream CBD also showed mild dilatation (calibre ~7.8 mm). The mural soft tissue appeared to be heterogeneously hyperintense on T2-weighted sequences and showed diffusion restriction. Triple phase contrast enhanced sequences showed late arterial enhancement in the mural soft tissue, with reduced enhancement on the porto-venous and delayed phases (Figure 1, Figure 2, Figure 3 and Figure 4).

**FIGURE 5:**
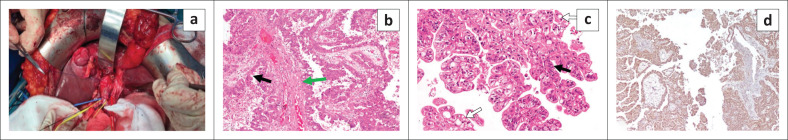
(a) The patient underwent open hilar resection with standard lymphadenectomy and right and left Roux-en-Y-cholangiojejunostomy. Papillary growth was seen at the primary confluence, extending into the right hepatic duct. (b) Low–power photomicrograph (Hematoxylin & eosin [H & E] stain; magnification 10×) of the resected specimen showing papillary projections (black arrow) with a central fibrovascular core (green arrow). (c) High-power photomicrograph (H & E stain; magnification- 20×) showing irregular papillae with features of high-grade dysplasia and hyperchromatic nuclei (black arrow); goblet cells are also seen (white arrow). (d) Positive immunohistochemistry staining (brown) for MUC2 and MUC5 using recombinant antibodies.

The patient subsequently underwent open hilar resection with standard lymphadenectomy. The right and left hepatic ducts were individually anastomosed to a jejunal loop by Roux-en-Y hepaticojejunostomy. Gross examination of the resected cyst revealed mucinous material. Histopathological analysis and Immunohistochemistry staining of the resected specimen confirmed the lesion to be an IPN-B (intestinal subtype, histological type 2 with high-grade dysplasia) restricted to the primary confluence and right hepatic duct, with tumour-free CBD, left hepatic duct and hepatic margins (Figure 5).

## Discussion

Intraductal papillary neoplasm of the bile duct is a rare intraductal preinvasive neoplasm, which is analogous to intraductal papillary mucinous neoplasm of the pancreas. Histologically, it is found to arise from the biliary tree stem cells in peribiliary glands, which can differentiate along the pancreatico-biliary, gastric, intestinal or oncocytic lines.^2,4,6^ The intestinal subtype expressing mucin core protein MUC2 and MUC5AC is more commonly found in the Asian population, while the pancreatico-biliary subtype expressing MUC1 is more frequent in the Western population and carries a higher risk of malignant conversion. Within the pancreatico-biliary histological type, the biliary type is more linked with malignant conversion, while the pancreatic subtype shows more mucin production.^2^

The disease is commonly found in men aged 40–80 years and patients often present with symptoms related to biliary obstruction caused by the tumour itself (upstream dilatation) or by the excessive mucin production (downstream dilatation).^2,7^ Within the Asian population, it most commonly involves the intrahepatic system (left lobe>right lobe), with risk factors including hepatolithiasis and Clonorchis infection.^2^

Morphologically, Ying et al.^8^ divided IPN-B into seven sub-types, depending on the presence of intraductal polypoidal mass and site of dilatation-

- An intraductal, polypoidal mass with minimal (type 1) or abundant (type 2) mucin production showing ectatic upstream biliary dilatation. Mucin production (type 2) was also associated with downstream biliary dilatation.
- No intraluminal polypoidal mass with abundant mucin production causing diffuse dilatation of upstream and downstream biliary radicles (Type 3 and 4). Type 3 showed a superficial spreading, cast-like mural thickness on resection, whereas Type 4 showed no evidence of any growth.
- Polypoidal intraluminal growth causing cystic, focal, aneurysmal dilatation of the intrahepatic (type 5) or extrahepatic biliary system (type 6). This morphology is considered typical of IPN-B and is found in the presented case as well.
- Infiltrating mass showing extraductal spread with biliary dilatation, upstream and/or downstream. This morphology is difficult to distinguish from mass forming, invasive cholangiocarcinoma. A large extraductal mass with evidence of invasion of adjacent organs is suggestive of malignancy.^9^

Depending on the histological grade, IPN-B can be further divided into (1) type 1 including mild to intermediate grade dysplasia and (2) type 2 including high-grade dysplasia.^2,5^

At ultrasound examination, the intraductal mass is commonly seen as a hypo or hyperechoic mass inside anechoic-dilated bile ducts.^10^ Mucin cannot be differentiated from bile on ultrasound. Contrast-enhanced ultrasound cannot help us in distinguishing benign, preinvasive lesions from malignant lesions. At CT, the solid components appear hyperattenuating compared with the surrounding hepatic parenchyma. At MRI, the solid component appears to be hypointense on T1 and hyperintense on T2-weighted images. Multiphasic contrast-enhanced scans are most important in differentiating IPN-B from other intraluminal neoplastic and nonneoplastic aetiologies. The solid component of IPN-B tumours tends to show contrast uptake in the late arterial phase, with reduced enhancement on porto-venous and delayed phases due to lack of a fibrous component.^2,11^ This is consistent with the findings in this case report. Intraductal mucin may be seen as string-like filling defects on MRCP or on the hepato-biliary phase of contrast-enhanced MRI. Endoscopic pancreatico-cholangiography (ERCP) helps in the confirmation of the diagnosis by visualisation of mucin discharge from the major papilla and obtaining a biopsy.^12,13^

Given the type 5 morphology of the lesion in the presented case, important differentials and imaging characteristics for consideration are presented in Table 1.

**TABLE 1:** Differential diagnoses of intrahepatic, intraductal solid-cystic tumours.

Differentials	Differentiating parameters	Differentiating features
Non-neoplastic filling defects like calculi^2^	Signal intensity on T2W-MRI Contrast enhancement Diffusion restriction	Calculi – Marked hypointensity on T2W images, no diffusion restriction and no contrast enhancement
**Hepatobiliary cysts** ^2,7^		
Non-neoplastic cysts like simple peribiliary hepatic cysts, choledochal cysts (focal Caroli’s disease) and hydatid cysts	Solid, enhancing components	Non-neoplastic cysts do not show internal solid enhancing components
Neoplastic cystic lesions like mucinous cystic neoplasm (MCN) (cystadenoma and cystadenocarcinoma)	Morphology and histopathology	MCN – large, multiseptated, multilocular cystic lesion with no connection to the biliary tree; Histopathology reveals sub-epithelial ovarian stroma
**Other neoplastic intraductal masses^2^**		
Intraductal cholangiocarcinoma (ID-CC)	Morphology of tumour and biliary system, site, enhancement characteristics	ID-CC — intraductal, solid mass, usually extrahepatic, showing only upstream biliary dilatation, and delayed persistent enhancement^14^
Hepatocellular carcinoma with bile duct invasion (HCC-BDI)	Surrounding liver	HCC-BDI – contiguous extraductal liver parenchymal mass with expansile luminal extension; backdrop of cirrhosis^15^
Biliary metastasis	Evidence of primary tumour	Contiguous extraductal liver parenchymal mass; most common source of primary colon^15^

MRI, magnetic resonance imaging.

Since a malignant component can be found in nearly 33% – 80% of cases of IPN-B, the primary management is surgical resection with a tumour-free margin. Depending on the extent and site of the lesion, surgery can be either partial hepatectomy with CBD resection (intrahepatic lesions) similar to the case presented or isolated CBD resection with a hepatico-jejunostomy (extrahepatic lesions).^2^

The 5-year survival rate of IPN-B has been found to be as high as 80.9%, with the surgical outcome mainly dependent on the tumour site, size and obtaining a free surgical resection margin (R_0_).^16,17^ Although the 5-year survival rate is relatively better than cholangiocarcinoma, due to the high rate of recurrence, long-term follow-up with MRCP is advised.^18^

## Conclusion

Intraductal papillary neoplasm of the bile duct is a relatively rare but important differential to consider while assessing a case of cystic biliary dilatation and biliary intraductal masses. Characterised by mucin production causing upstream and downstream biliary dilatation, patients usually present with symptoms of biliary obstruction. It is important to rule out malignant mimics like intraductal cholangiocarcinoma, which have a worse prognosis, more radical management and poorer patient outcome.
